# Cognitive and Psychiatric Relevance of Dynamic Functional Connectivity States in a Large (N>10,000) Children Population

**DOI:** 10.21203/rs.3.rs-3586731/v1

**Published:** 2024-01-12

**Authors:** Zening Fu, Jing Sui, Armin Iraji, Jingyu Liu, Vince Calhoun

**Affiliations:** Georgia Institute of Technology, Emory University and Georgia State University; Georgia State University; Georgia State University; Georgia State University

**Keywords:** children, dynamic functional connectivity state, cognition, psychopathology, large-scale analysis

## Abstract

Children’s brains dynamically adapt to the stimuli from the internal state and the external environment, allowing for changes in cognitive and mental behavior. In this work, we performed a large-scale analysis of dynamic functional connectivity (DFC) in children aged 9 ~ 11 years, investigating how brain dynamics relate to cognitive performance and mental health at an early age. A hybrid independent component analysis framework was applied to the Adolescent Brain Cognitive Development (ABCD) data containing 10,988 children. We combined a sliding-window approach with k-means clustering to identify five brain states with distinct DFC patterns. Interestingly, the occurrence of a strongly connected state was negatively correlated with cognitive performance and positively correlated with dimensional psychopathology in children. Meanwhile, opposite relationships were observed for a sparsely connected state. The composite cognitive score and the ADHD score were the most significantly correlated with the DFC states. The mediation analysis further showed that attention problems mediated the effect of DFC states on cognitive performance. This investigation unveils the neurological underpinnings of DFC states, which suggests that tracking the transient dynamic connectivity may help to characterize cognitive and mental problems in children and guide people to provide early intervention to buffer adverse influences.

## Introduction

Functional connectivity (FC) derived from functional magnetic resonance imaging (fMRI), which quantifies the temporal statistical dependencies between functional activation in different brain regions, provides the most insightful correlates between personal behavior and the brain [1]. FC is traditionally considered a static and trait-level characteristic of individuals [2–4]. For example, FC strength alterations have been linked to changes in cognitive performances [5, 6], sex-related differences [7], and a wide range of brain diseases [8–11]. However, this implicit assumption of temporal stationarity of FC has been challenging. A growing body of research believes that the dynamic nature of the brain enables changes in cognition and behavior that allow an individual to adapt to a complex environment [12, 13]. Dynamic FC (DFC) approaches, which consider the temporal fluctuations in FC within a single scan [14, 15], track transient brain organization in a finer timescale. DFC can quantify the time-varying information in cognitive states and task demands [16], providing insights into how FC dynamics are related to cognition and psychiatric problems [17, 18]. These findings suggested the great potential of tapping into the relation between FC dynamics and individuals’ behavior, especially for children, as cognitive problems and mental health are critically important for children at this stage of brain development.

Developmental cognitive neuroscience that investigates the associations between neuroimaging and cognitive ability is a rapidly growing field in recent decades [4, 20]. Many functionally-integrated neural networks emerge in early childhood [4], which is responsible for the maintenance of cognitive functioning and mental behavior [21–23]. Recent studies have found that resting-state FC is a reliable indicator of cognitive performance in children [24, 25], and impairments in FC networks might lead to the worsening of cognitive functioning in early childhood. Mental diseases often first manifest during childhood and adolescence [26, 27], and alterations of FC in neurocognitive networks have been shown to underlie dimensional indices of psychopathology, including internalizing and externalizing psychopathology [23, 28, 29]. Abnormal FC has been identified in children with different brain disorders, such as Attention Deficit Hyperactivity Disorder (ADHD) [11], reflecting that dimensions of psychopathology in children may be related to the derangement of functional circuits. Individual differences in anxiety are highly correlated with FC between the amygdala and distributed brain systems in children at 7 to 9 years old [30]. Robust correlations have also been characterized between default-mode network FC with higher neurodevelopmental dimension scores measured by the Child Behavioral Checklist (CBCL) [28]. More recently, studies have demonstrated the utility of DFC for understanding functional neurodevelopment in children. Marusak et al. have found that the occurrence of a DFC state is related to the content of self-generated thoughts in children [32]. By applying DFC analysis to 28 young children, a study found that the decreased DFC variance between the posterior cingulate cortex (PCC) and the right precentral gyrus is associated with the decline in social motivation and social relating [33].

Despite such progress, studies focusing on DFC in children have ignored the potential interactive relationships between mental health, cognitive development, and DFC. Mental health and cognitive performance in children might have an interactive relationship. For example, attention plays a critical role in cognitive and social development, and its deficits affect many children with various mental illnesses, such as ADHD [34]. In addition, previous work has used a relatively small number of children and failed to demonstrate the test-retest reliability of the DFC findings, indicating the need for a robust large-scale study such as the present one that included more than 10,000 participants. A comprehensive exploration of the DFC in children can help to draw a full picture of brain functional dynamics in early life, which might improve our understanding of the neural mechanisms underlying the association between functional brain organization and behavior formation in children.

In this study, we aimed to establish robust DFC patterns in children using large-scale neuroimaging data from Adolescent Brain Cognitive Development (ABCD). This longitudinal and multi-sessional data with a large number of participants provides an unprecedented opportunity to examine reliable DFC and its associations with cognitive and mental behavior in children [35]. We applied a fully automated independent component analysis (ICA) framework to probe the reoccurring DFC patterns during the resting-state. We hypothesized that children’s resting-state FC is highly fluctuated within a single scan, with reoccurring connectivity patterns underlying different information transfer abilities. The connectivity patterns, namely as DFC states, are reproducible across scans and sessions. Cognitive or mental problems in children might be related to the time they spend in distinct DFC states. A recent study found that mental health mediates the relationships between brain structure and sleep condition [37]. They also found that sleep condition mediates the relationship between brain structure and cognition. These findings suggest that the covariance between brain imaging and cognition is partially explained by mental health. We therefore hypothesized that the associations between DFC and cognitive performance might be related to psychiatric problems.

## Materials And Methods

### Participants And Preprocessing

This study was based on release 3.0 of the Adolescent Brain Cognitive Development (ABCD) database downloaded from the National Institute of Mental Health Data Archive (NDA, https://nda.nih.gov/), containing over 11,800 children aged 9–11 years old (at the baseline), with two imaging sessions and multiple scans within each session. Parents’ full written informed consent and children’s assent were obtained under protocols approved by the Institutional Review Board (IRB). We preprocessed the raw resting-state fMRI data using a combination of the FMRIB Software Library (FSL) v6.0 (FSL) toolbox and Statistical Parametric Mapping (SPM) 12 toolbox. Data quality control (QC) was performed on the preprocessed resting-state data for selecting subjects with good normalization to the MNI standard space for further analysis. Details of the preprocessing and QC are provided in the supplementary materials.

### Cognitive And Mental Health Assessments

To investigate the relationships between DFC and comprehensive cognitive functions, we used the uncorrected raw scores from the National Institutes of Health Cognition Battery Toolbox (NIHTBX) [38, 39] to evaluate the cognitive performance of children. We used the raw scores from the parent-rated Child Behavior Checklist (CBCL) to investigate the associations between DFC and children’s mental health. The reasons for choosing these assessments are provided in the supplementary materials. We also validated our results using other cognitive and mental health batteries, including the ABCD Pearson Scores (PS: abcd_ps01) as the measurements of cognition and the ABCD Parent Diagnostic Interview for DSM-5 Full (KSADS-5: abcd_ksad01) and ABCD Parent General Behavior Inventory-Mania (PGBI: abcd_pgbi01) as the measurements of mental health.

### Neuromark Framework To Extract Regions Of Interest

We implemented the Neuromark framework via the group independent component analysis (ICA) of fMRI toolbox [40]. Neuromark is a unified ICA framework that can extract comparable neuroimaging features across subjects, scans, and datasets. It adopted two independent healthy datasets to create a group of component templates as regions of interest (ROIs). The component templates were then set as references in a spatial-constrained ICA for the estimation of single-scan components and corresponding TCs for the ABCD dataset. Details of the Neuromark are introduced in the supplementary materials.

### Dynamic Functional Connectivity And Clustering

TCs of ROIs of each scan underwent additional post-processing to remove the remaining noise. These procedures included: 1) detrending linear, quadratic, and cubic trends, 2) multiple regression of six realignment parameters and their derivatives, 3) removal of detected outliers, and 4) band-pass filtering with a cutoff frequency of 0.01 Hz-0.15 Hz. DFC was estimated with a sliding window approach with a window size of 40 repetition times (TRs). A tapered window, created by convolving a rectangle (width = 40 TRs = 32 s) with a Gaussian (σ = 3 TRs), was used for segmenting the TCs.

We performed k-means clustering with Euclidean distance on the DFC estimates to identify reoccurring FC patterns. The optimal number of states was estimated using the elbow criterion [41]. The number of clusters was *k* = 5, within a reasonable range consistent with previous DFC studies [42–44]. To show the reliability of our results, we replicated the findings by using a different number of clusters (3 ~ 6) in the k-means clustering. The fractional rate is calculated as the percentage of windows clustered to each brain state to measure the state occurrence. Additional reliability analyses [45, 46] based on the DFC states were performed to demonstrate the stability of the subject-specific DFC states. We also implemented two null models, Vector autoregressive (VAR) model [47, 48] and the phase randomization model [49], to demonstrate that the fluctuations in DFC reject the null hypothesis of stationary.

### Associations Between DFC States And Children’s Behavior

A linear mixed-effect model (LMM) was implemented to investigate the associations between the state occurrence and the cognition/psychiatric problems. The ABCD data contain related data at sites and within families due to twins and siblings. The LMM can model family nested within the site to take account of the correlated observations. In this model, the fractional rate of each brain state was modeled as the dependent variable, while each cognitive/mental health score was modeled as a fixed effect. Age, gender, race, height, and weight were the confounding effects, modeled as other fixed effects. The family structures and sites were modeled as random effects [37]. These covariates are potential confounding effects that are suggested to be regressed in the previous ABCD studies [37, 51]. The correlation r-value, t-statistic, and effect size Cohen’s d were calculated for each association analysis to reflect the relationship between a DFC state and a score. The results were corrected by false discovery rate (FDR) correction [52]. Results were also replicated by controlling the head motions and excluding the large head motions subjects.

### Test-retest Reliability

We first split the discovery data in half, one half included data from randomly picked 10 sites, and the other half included data from the remaining 11 sites. We performed the identical analysis for each half and matched the DFC states to the state centroids obtained using data from all 21 sites. We then replicated the associations between DFC states and cognitive/mental scores. Additionally, we repeated the analysis by selecting the same number of subjects from each site. 294 subjects were randomly selected for each site, resulting in a total of 6174 subjects for the analysis. We performed the same analysis to explore the relationships between DFC states and cognition/mental scores.

To further show the test-retest reliability of our findings, we ran an independent analysis using the second scan from the baseline session. To show that brain maturation through adolescence will not influence the reoccurring DFC states and their associations with cognition and mental health, we also replicated the results using two different scans from the second-year session. The resulting state centroids were matched to the centroids from discovery data (first scan from the baseline). The associations between DFC states and cognition/mental scores were investigated using LMM.

### Mental Health Mediates The Relationship Between DFC States And Cognition

Our hypothesis investigated the relation of brain dynamics with psychiatric problems and cognitive performance. We performed a standard mediation analysis using the Mediation Toolbox (https://github.com/canlab/MediationToolbox). We used a standard three-variable path model. The fractional rate of a DFC state was the independent variable, and the cognitive score was the dependent variable. The mental problem score was the mediator. Confounding variables (including age, gender, race, height, and weight) were regressed in the mediation model. The significance of the mediation was estimated by a bias-corrected bootstrap approach with 10000 random samplings. The mediation effect is a measure of association, which does not assume directionality aspects of these relationships.

### Graph Analysis Demonstrates Topological Difference Between DFC States

We calculated the graphic measures of highlighted DFC states and examined their differences between brain states. Node strength and efficiency were estimated based on the DFC matrix within each state using the brain connectivity toolbox (https://sites.google.com/site/bctnet/). For each subject, we averaged the DFC estimates assigned to each state to obtain the mean state-based DFC. Then we used the positive FC, negative FC, and absolute FC of the state-based DFC to construct three graphs. The graphic measures were calculated based on the three graphs and were compared between states using a pair-wise t-test for each ROI respectively.

## Results

### Brain Parcellation

We selected subjects with at least two good resting-state scans for the baseline or the second-year sessions. These criteria yield 10988 subjects with two good resting-state scans for the baseline session and 3622 subjects with two good resting-state scans for the second-year session. The first scan from the baseline session was the discovery data and the remaining three scans were the replication data. The basic demographics of the subjects are provided in Table I.

The Neuromark framework was applied to each scan, parcellating the brain into 53 meaningful components as the regions of interest (ROIs). The 53 ROIs were arranged into seven functional networks according to their anatomical and presumed functional properties. Specifically, there are five ROIs in the subcortical network (SC), two ROIs in the auditory network (AUD), nine ROIs in the sensorimotor network (SM), nine ROIs in the visual network (VS), 17 ROIs in the cognitive-control network (CC), seven ROIs in the default-mode network (DM), and four ROIs in the cerebellar network (CB).

### Highly Reproducible DFC States

**Figure 1:**
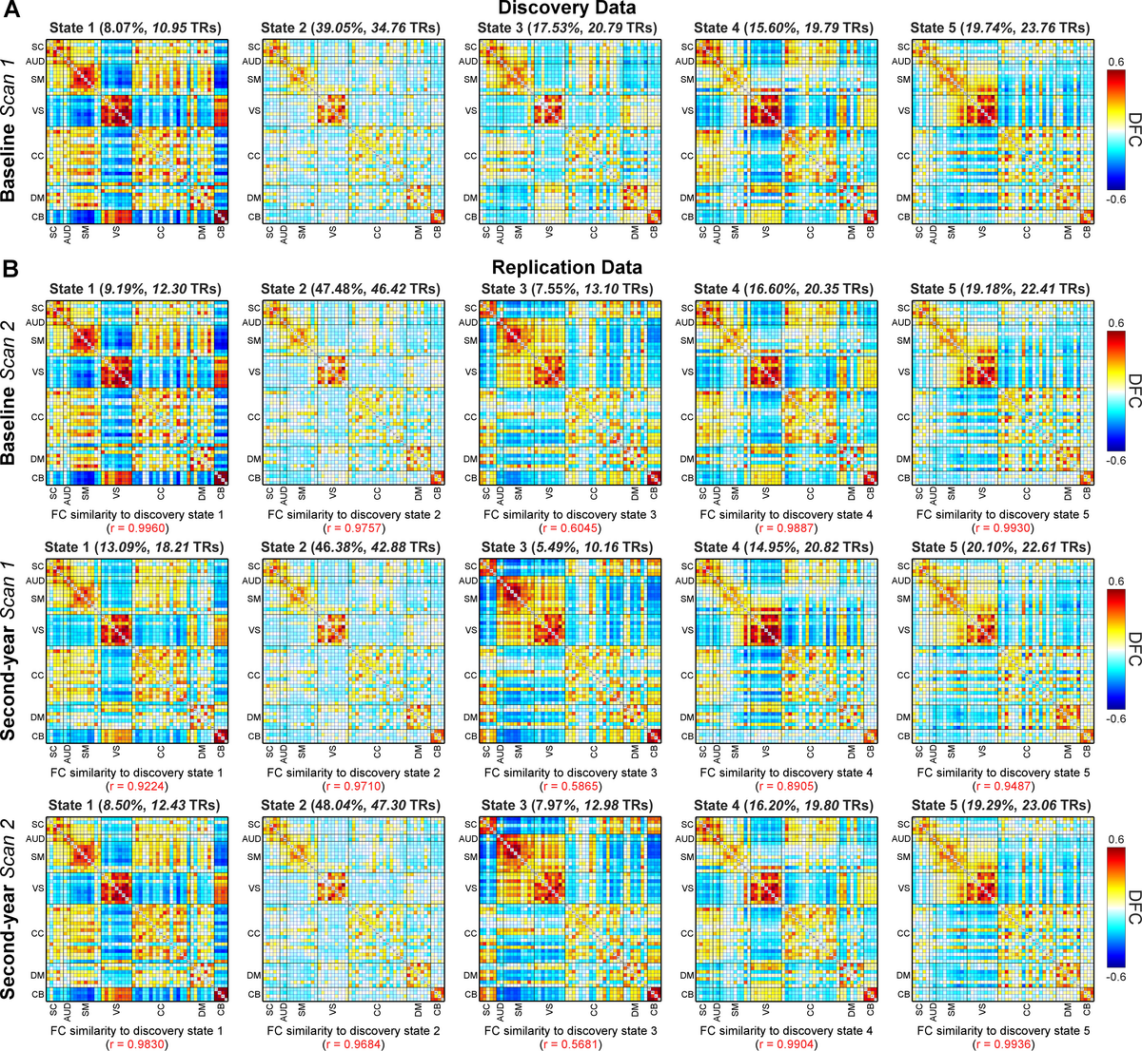
Reproducible brain states across resting-state scans and between longitudinal sessions. Five DFC states are identified by the k-means clustering for each scan, respectively. A) the FC patterns of DFC states for scan 1 of the baseline session (discovery data). B) the FC patterns of DFC states for replication data, including scan 2 of the baseline session, scan 1 of the second-year session, and scan 2 of the second-year session. Brain states are matched by evaluating the correlation coefficient between the centroid patterns. Four DFC states (columns 1, 2, 4, and 5) are highly replicable between the discovery and replication data (r > 0.8).

Figure 1 displays the centroid patterns of the k-means clustering results. The first row (Fig. 1A) shows the centroid patterns of each brain state for the discovery data and the second to fourth rows (Fig. 1B) show the centroid patterns for the replication data. The brain states from the replication data were matched to the brain states from the discovery data by evaluating the correlation between their centroid patterns. 4 out of 5 DFC states are highly replicated among scans with averaged r values > 0.8. State 1 from the discovery data has matched states from the replication data (r = 0.9960, 0.9224, and 0.9830, respectively). It shows strongly positive FC within the sensory networks (AUD, SM, and VS) and the most negative connectivity of SM network with VS and CB networks. It has the lowest occurrence across the five states (fractional rate [the percentage of windows that is assigned to a given state] is around 8% ~ 13% windows). State 2 is a sparsely connected state replicated across scans (r = 0.9757, 0.9710, and 0.9684) and has the highest occurrence during the resting-state (fractional rate is around 39% ~ 48% windows). State 4 shares similar FC patterns with state 1, but has weaker connectivity, especially within the SM network. State 5 is the only state with positive FC between SM and VS networks, positive FC between CC and CB networks, and negative FC between VS and CB networks.

### DFC States Are Associated With Children’s Cognitive Performance

**Figure 2:**
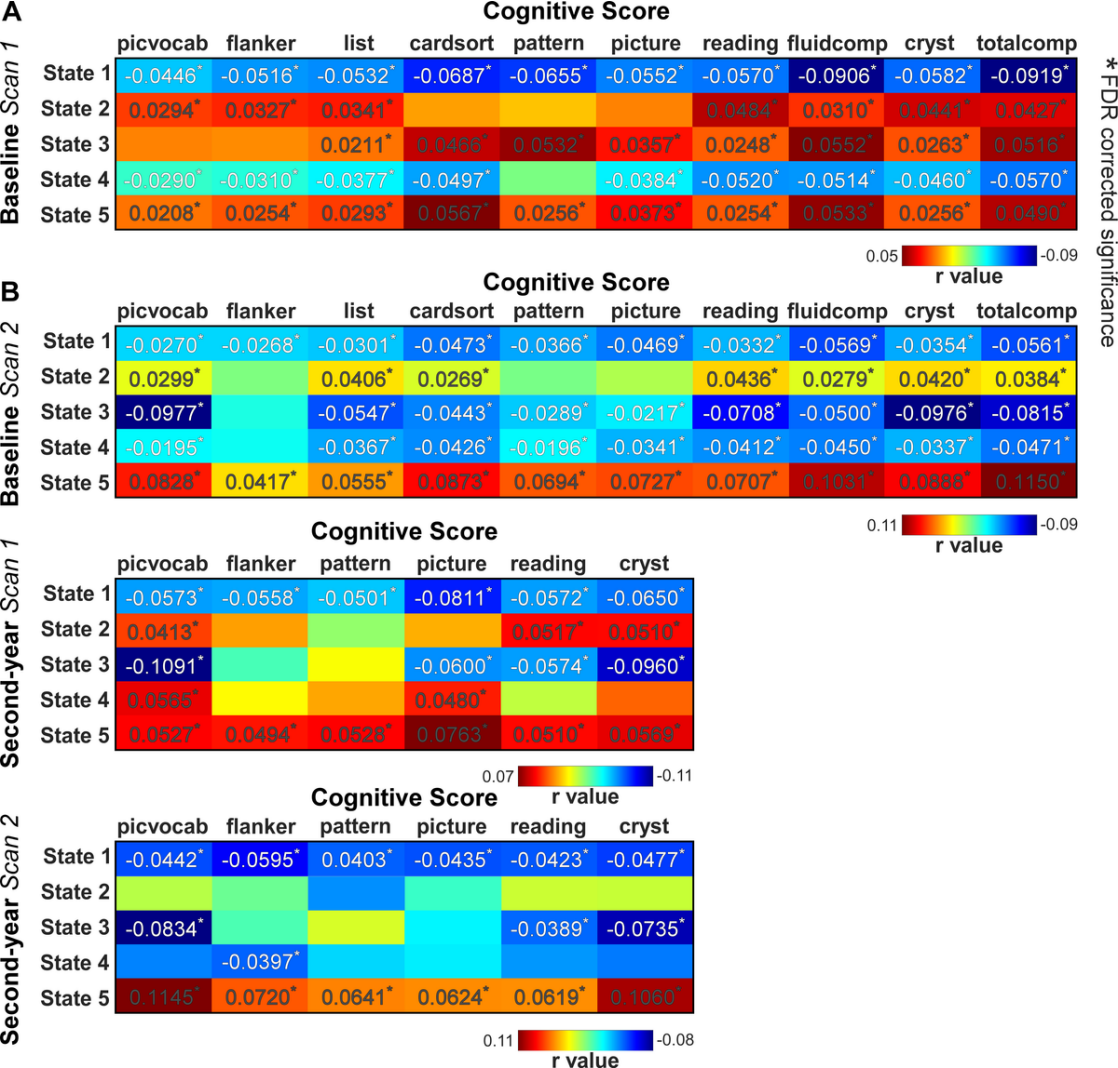
DFC states are correlated with children’s cognitive performance. A) Significant correlations between DFC states and cognitive scores are identified using the discovery data (FDR corrected, q < 0.05). Strongly connected DFC states are negatively associated with cognitive scores and sparsely connected DFC states are positively associated with the cognitive scores. Children with good cognitive performance tended to have fewer occurrences in the strongly connected states and more in the weak connected states. B) Significant correlations between DFC states and cognitive scores are replicated using scan 2 from the baseline session, and two scans from the second-year session. Reproducible brain states show consistent associations with children’s cognitive performance.

The cognitive scores were negatively correlated with the fractional rates of the DFC states with strong within- and between-network connectivity (e.g., states 1 in Fig. 2A). 10 out of 10 cognitive scores measured by the National Institutes of Health Cognition Battery Toolbox (NIHTBX) were negatively correlated with the fractional rate of state 1, with correlation r values ranging from − 0.0446 to −0.0919 (False discovery rate [FDR] corrected, q < 0.05). The cardsort score, fluid composite score, and total composite score were the score most significantly negatively correlated with the fractional rate of state 1 (r = −0.0687, Cohen’s d = −0.1378, p = 7.97×10^−13^; r = −0.0906, Cohen’s d = −0.1820, p = 4.52×10^−21^; and r = −0.0919, Cohen’s d = −0.1875, p = 1.30×10^−21^). In contrast, the cognitive scores were positively correlated with the fractional rates of the DFC states with weak and sparsely connected patterns (e.g., state 5). 10 out of 10 cognitive scores were positively correlated with the fractional rate of state 5, with correlation r values ranging from 0.0208 to 0.0567. Similarly, the cardsort score, fluid composite score, and total composite score were the scores most significantly positively correlated with the fractional rate of state 5 (r = 0.0567, Cohen’s d = 0.1136, p = 3.44×10^−9^; r = 0.0553, Cohen’s d = 0.1068, p = 3.13×10^−8^; and r = 0.0490, Cohen’s d = 0.0981, p = 3.72×10^−7^). The associations between DFC states 1 and 5 and cognitive scores were replicated using baseline scan 2 and the scans from the second-year session (Fig. 2B).

### DFC States Are Associated With Children’s Mental Health

**Figure 3:**
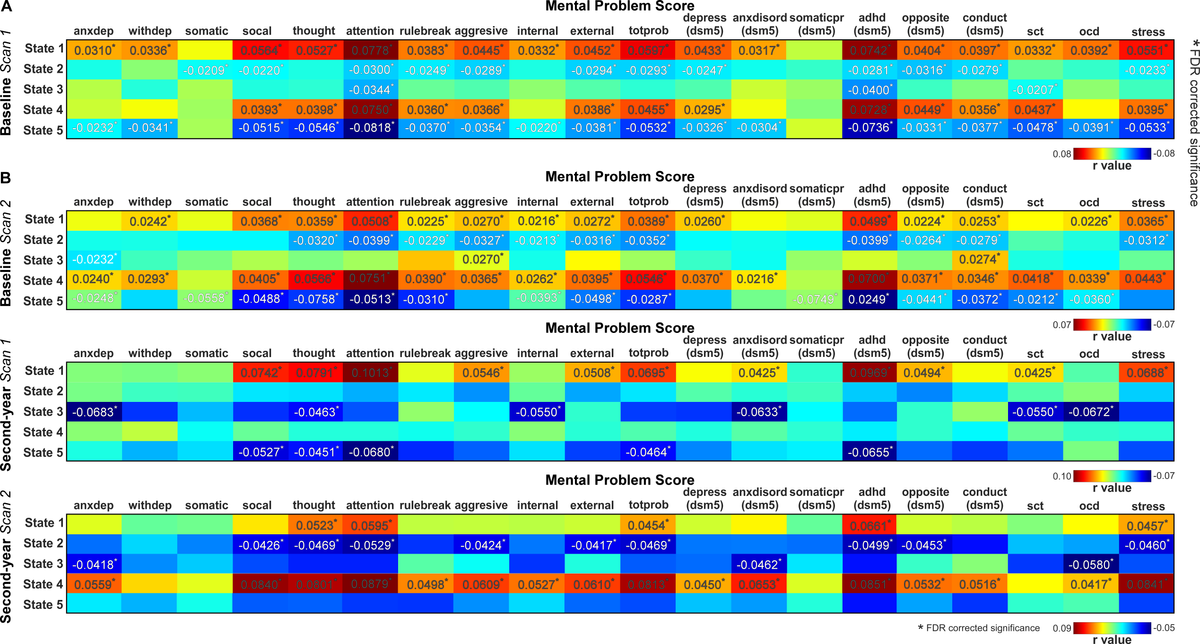
DFC states are correlated with children’s mental health. A) Significant correlations between DFC states and psychiatric scores are identified using the discovery data (FDR corrected, q < 0.05). Strongly connected DFC states are positively associated with the psychiatric scores and sparsely connected DFC states are negatively associated with the psychiatric scores. Children with high mental problem scores tended to have more occurrences into the strongly connected states and fewer occurrences into the weak connected states. B) Significant correlations between DFC states and psychiatric scores are replicated using scan 2 from the baseline session, and two scans from the second-year session. Reproducible DFC states show consistent associations with children’s mental health.

The psychopathological measures of children were significantly correlated with the fractional rates of DFC states. Interestingly, in contrast to the cognitive measures, children’s mental problem scores were positively correlated with the strongly connected states (e.g., state 1 in Fig. 3A) and negatively correlated with the sparsely connected states (e.g., state 5 in Fig. 3A). Specifically, 18 out of 20 psychiatric scores measured by the CBCL scores were positively correlated with the fractional rate of state 1, with correlation r values ranging from 0.0310 to 0.0778 (FDR corrected, q < 0.05). These mental problem scores were negatively correlated with the fractional rate of state 5, with correlation r values ranging from − 0.0232 to −0.0818. Among these scores, the attention score and the Diagnostic and statistical manual of mental disorders (DSM-5) ADHD score were the most significantly correlated with the fractional rate of DFC states (**state 1:** r = 0.0778, Cohen’s d = 0.1561, p = 3.36×10^−16^; r = 0.0742, Cohen’s d = 0.1487, p = 7.36×10^−15^; **state 5:** r = −0.0818, Cohen’s d = −0.1641, p = 9.58×10^−18^; r = −0.0736, Cohen’s d = −0.1476, p = 1.16×10^−14^). The psychiatric relevance of DFC states was validated using baseline scan 2, and two scans from the second-year session (Fig. 3B). The fractional rate of state 1 was positively associated with the mental problem scores, and the fractional rate of state 5 was negatively associated with the mental problem scores.

**Figure 4:**
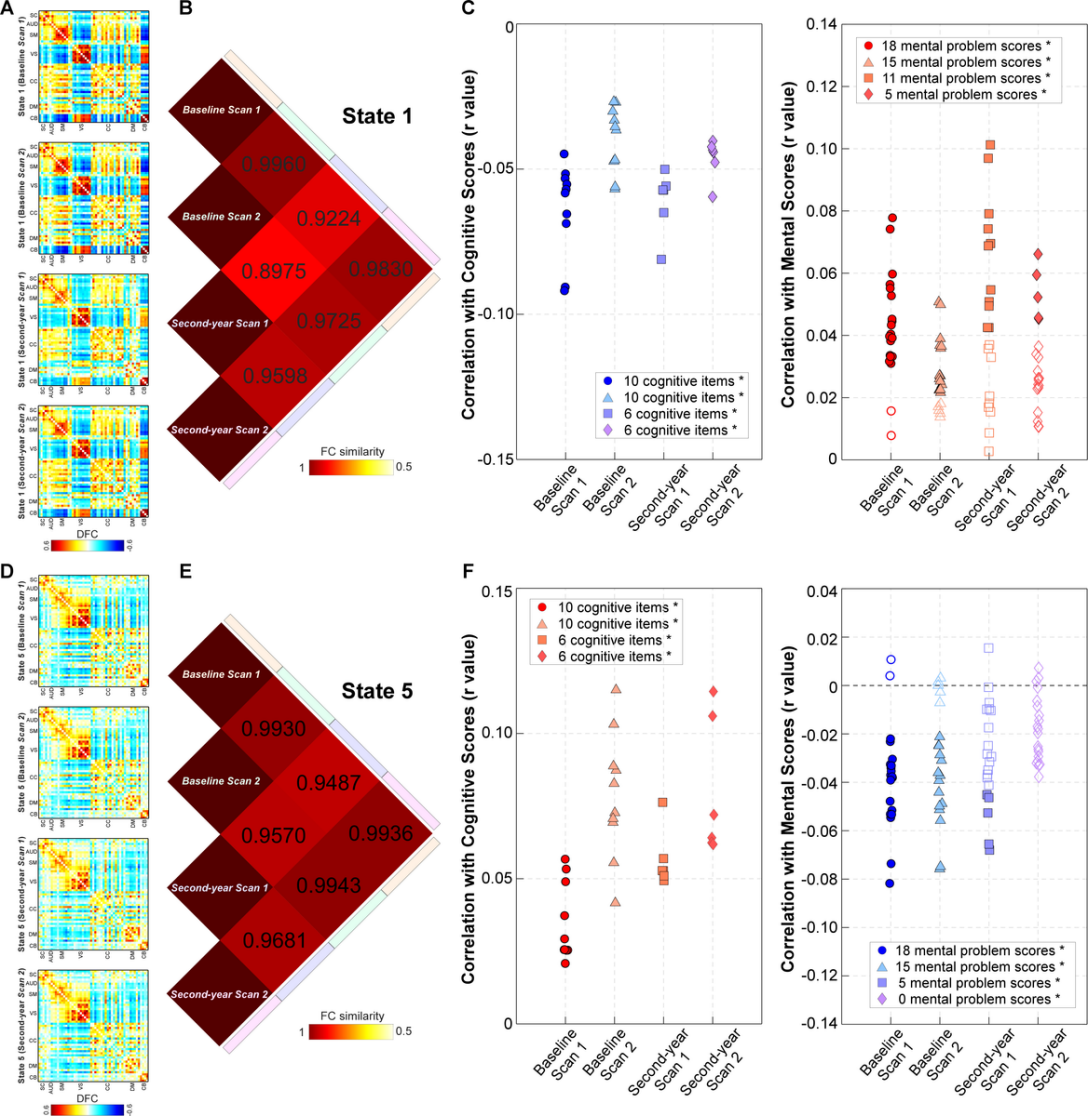
Two DFC states show robust associations with children’s cognitive performance and mental health. A) Brain state 1 with strongly positive and negative connectivity patterns is reproducible across multiple scans. B) Reproducible brain states show high inter-correlations > 0.85. C) The fractional rate of the strongly connected states shows negative correlations with cognitive scores and positive correlations with mental problem scores. D) Brain state 5 with sparse connectivity patterns is reproducible across multiple scans. E) Reproducible brain states show high inter-correlations > 0.9. F) The fractional rate of the weakly connected states shows positive correlations with cognitive scores and negative correlations with mental problem scores.

We highlight two DFC states that show robust associations with children’s cognition and mental health. The first state is DFC state 1, which was reproducible across multiple scans (Fig. 4A) with inter-correlations higher than 0.89 (Fig. 4B). The fractional rate of this state was negatively correlated with children’s cognitive performance and positively correlated with children’s mental problem scores (Fig. 4C). The second state is DFC state 5 (Fig. 4D), which was also replicable across scans (inter-correlations higher than 0.94). The fractional rate of this state was positively correlated with the cognitive scores and negatively correlated with the mental problem scores (Fig. 4F).

### Mental Health Mediates Effects Of DFC States On Cognition

**Figure 5:**
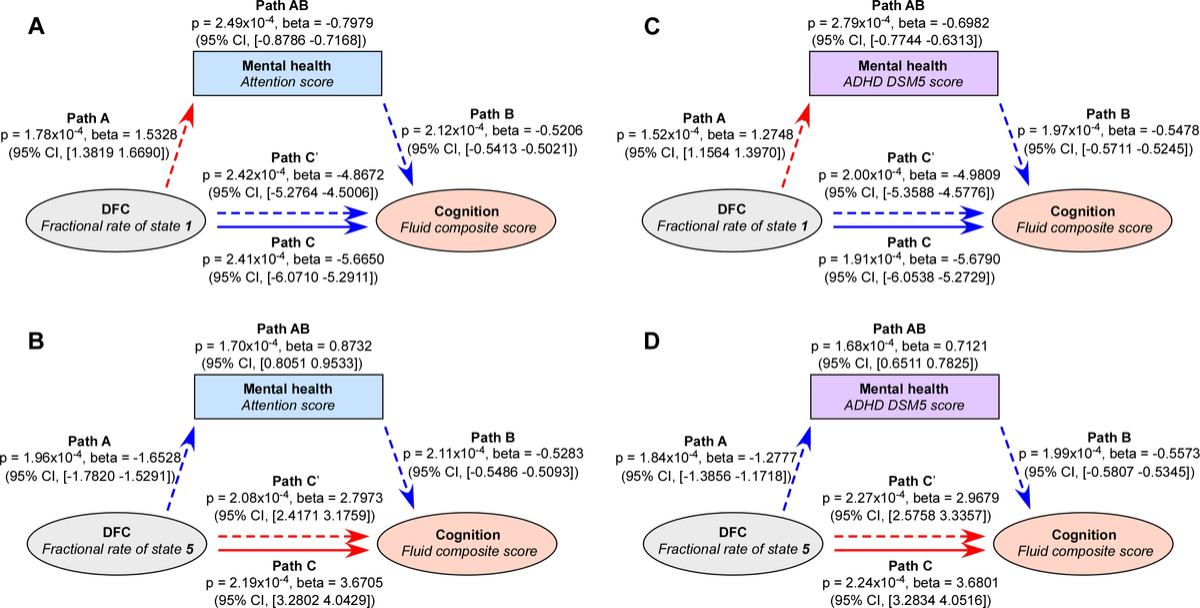
The mediation effects among DFC states, children’s cognition and mental health. A) The mediation implemented by attention problems from the state 1 on the fluid composite score is significant (beta = −0.7979, p = 2.49 × 10–4, 14.08% of the total effects). B) The mediation implemented by attention problems from the state 5 on the fluid composite score is significant (beta = 0.8732, p = 1.70 × 10–4, 23.79% of the total effects). C) The mediation implemented by ADHD scores from the state 1 on the fluid composite score is significant (beta = −0.6982, p = 2.79 × 10–4, 12.29% of the total effects). D) The mediation implemented by ADHD scores from the state 5 on the fluid composite score is significant (beta = 0.7121, p = 1.68 × 10–4, 19.35% of the total effects). The indirect paths A, B and AB show that mental health mediates part of the effect of DFC states on the fluid composite score. Path A: effect of the fractional rate of DFC states on the mediator, mental health scores; Path B: effect of the mediator on cognitive performance; Path C’: direct effect of the fractional rate of DFC states on cognitive performance; Path AB: effect of DFC states on cognition explained by the mental health problems.

The baseline data was used for the mediation analysis. Similar to [37, 53], the mediation analysis in our study aimed to show how much of the correlation between DFC states and cognition is related to mental health. This effect does not prove causality. We selected the attention score and DSM-5 ADHD score which were the most strongly correlated with the DFC states as the mediators. The fractional rate of DFC states is the independent variable, and the fluid composite score is the dependent variable. The mediation analysis shows that the attention score mediated the effect of the occurrence of state 1 on the fluid composite score (Fig. 5A). The attention also mediated the effect of the occurrence of state 5 on the fluid composite score (Fig. 5B). Similar results were found for the DSM-5 ADHD score. The ADHD score mediated the effect of the occurrence of state 1 on the fluid composite score (Fig. 5C). In addition, the ADHD score mediated the effect of the occurrence of state 5 on the fluid composite score (Fig. 5D). We also identified similar mediation effects using other NIHTBX scores as the dependent variable and other CBCL scores as the mediator (contributing 0.72% ~ 51.98% of the total effect size).

### DFC States Show Distinct Network Topologies

**Figure 6:**
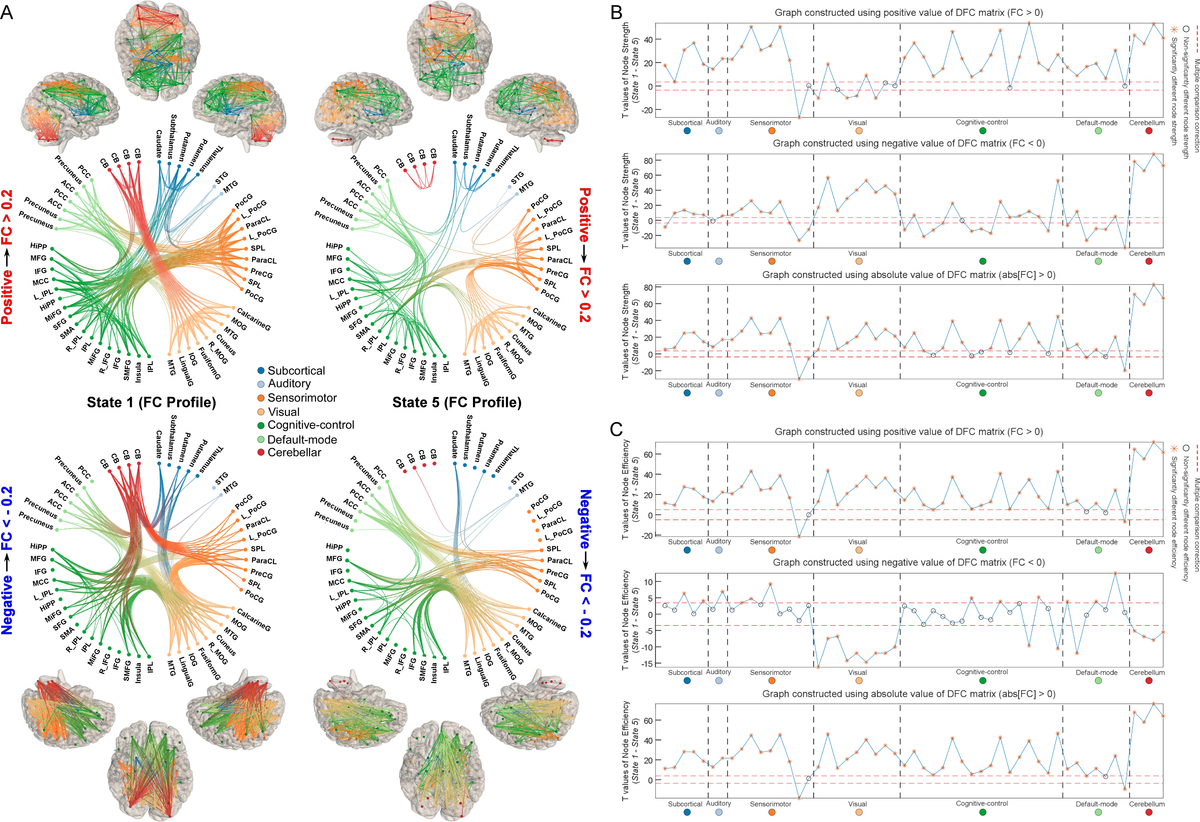
FC profile of DFC states and graph analysis on the centroid topologies. Graph is constructed using positive FC, negative FC or absolute FC respectively. A) The functional connectogram and brain mapping of FC centroids of states 1 and 5. FC of states’ centroids are divided into positive and negative values and thresholded to retain only strong connectivity for displaying (|FC| > 0.2) Pair-wise t-test results on node connectivity strength of ROIs between state 1 and state 5. C) Pair-wise t-test results on local efficiency of ROIs between state 1 and state 5.

Figure 6A displays the functional connectogram and brain mapping of FC centroids of states 1 and 5. We calculated node strength and efficiency via graph analysis [54]. The pair-wise t-test results of node strength are displayed in Fig. 6B. For the positive graph, most ROIs had larger node strength in state 1 (compared to state 5, FDR corrected, q < 0.05), except for those ROIs from VS network. For the negative graph, ROIs from VS and CB networks had larger node strength in state 1, while superior parietal lobule (SPL) and PCC had larger node strength in state 5 (FDR corrected, q < 0.05). The results for the absolute graph show that most ROIs had larger node strength in state 1, while SPL and PCC had larger node strength in state 5.

We further found that state 1 had significantly higher global efficiency than state 5 (p < 1.0 × 10^−100^). DFC states show different local efficiency patterns across ROIs as well. For the positive graph, most of the ROIs had higher local efficiency in state 1, especially for those ROIs from CB. However, interestingly, SPL and PCC had higher local efficiency in state 5. For the negative graph, ROIs from VS and CB networks had higher efficiency in state 5, while the other networks had higher efficiency in state 1. The results for the absolute graph are similar to the results for the positive graph, where only SPL and PCC had higher local efficiency in state 5.

## Discussion

### Robust DFC In Children

Given the dynamic nature of the brain, there is a strong consensus that dynamic patterns in fMRI FC likely have a neuronal origin, and as such, may engender a new understanding of brain organization [47, 55–57]. Our work is the first study that identified robust DFC states in a large (N > 10,000) children population, which are highly replicable across scans and between longitudinal sessions. In line with the findings in adult subjects, state 2 has the most sparsely connected FC patterns that resemble the static resting-state networks [15], accounting for the most time in the resting-state (> 39% of all windows). This state might signify the average of the additional conditions that are not sufficient to be separated [15]. State 1 shows the most coupling and antagonism within and between networks. Of note, while DM ROIs show weak or negative FC with the SM and CC systems in the other states, this state has strong positive FC between DM and SM/CC networks. This state challenges the notion of a stable DM network, as the DM regions have significant covariation with the frontal and sensorimotor cortex [15]. In addition, this state also shows strong couplings between VS and CB networks. Such a temporal covariation between visual and cerebellum systems might potentially resolve the confusion and ongoing discussion regarding the organization of cerebrocerebellar circuits in human brains [61, 62]. Specifically, the visual cortex can be temporarily represented in the cerebellum, reflecting the communication between the cerebral cortex and cerebellum via polysynaptic circuits [62]. Furthermore, note that SM ROIs that are typically uncorrelated with the VS ROIs exhibit different behavior in state 1 and state 5, showing strong positive and negative FC with VS network. These two states might capture the departure from the typical resting-state networks, in which brain regions form new functional interactions for a short time [63].

DFC state 3 shows the most distinct FC patterns across scans. We speculate that this state might not be frequent enough to be captured in baseline scan 1. This state shows thalamocortical antagonism which is a signature of loss of arousal in the resting-state [64]. Our previous work has shown that the DFC state with thalamocortical anticorrelation is associated with reduced EEG alpha power and increased delta and theta power, possibly reflecting the reduced vigilance in light sleep [58]. Along with previous investigations of FC dynamics [14, 58, 65], our results indicate that characterizations of FC from the dynamic perspective can provide finer topological mappings of the brain functional organization, as some meaningful network interactions can only be shaped by the short time scale over which FC is measured [66].

### DFC States And Cognitive Development In Children

The graph analysis shows that compared to state 1, state 5 has significantly higher PCC node strength and efficiency. This is partially consistent with a previous finding that the more time participants allocated to a dynamic state with strong DM FC, the better their performance was on executive functioning [67]. Although the precise functions of DM are still far from understood, brain regions comprising DM are largely involved in autobiographical memory, self-referential processes, and social cognitive processes [68, 69]. DM connectivity plays an essential role in human cognition and memory processing [70, 71]. However, there are still controversial findings on the relationship between DM FC and cognition. Some reported significant associations between changes in DM FC and cognitive impairment [72, 73] while others found no relationship between them [74]. Our result might explain this discrepancy by pointing out that, 1) cognition might have opposite relationships with different DM regions, and 2) cognition might be associated with the occurrence of states with different network topologies, rather than the constant FC. Previous work found that DM undergoes significant developmental changes in FC through adolescence but these changes are not uniform across all DM nodes [75]. Our result might provide further evidence to support this argument by showing that the FC of different DM regions can have different relationships with children’s cognition.

State 1 and state 5 also show significant differences in cerebellum-involved between-network FC. State 1 is the only state with strongly negative FC between the cerebellum and many other networks, including SC, SM, CC, and DM. As a pure motor system previously, the cerebellum has now come to be recognized for its participation in complex cognition, including social and emotional functions [76, 77]. The cerebellum receives information from the sensorimotor system, which might help develop general mechanistic accounts of cerebellar prediction and error processing in cognition [78]. Previous work also shows the contribution of cerebellar regions to different states of all investigated large-scale cortical networks [60]. We speculate that the entering of a DFC state (state 1) with negative FC might cause decreased information flow between the cerebellum and sensorimotor systems, which further results in temporal constraints on the cognitive capabilities of the cerebellum [78]. In contrast, the entering of a DFC state (state 5) with strong cerebellar between-network FC can enlarge these capacities and therefore promote better cognitive performance.

### DFC States And Mental Health In Children

Existing literature has provided much evidence implying the associations between DFC states and mental health. For example, schizophrenia patients with cognitive declines spent more time in a DFC state with strong sensory network connections [79]. In another study, Kim and colleagues reported an increased occurrence of a DFC state with strong within-network FC and negative between-network FC in Parkinson’s disease, which was not detected using static analyses [80]. In line with these findings, our results demonstrate that the DFC states can be related to mental behavior even in early childhood. Among all the psychiatric scores, the attention score and ADHD score show the most significant correlations with the DFC states. ADHD is a common childhood disorder characterized by pervasive expressions of inattention and hyperactivity [81]. We found that children with higher attention problem scores and ADHD scores tend to have more occurrence in the DFC state 1, which has higher node efficiency in the frontal gyrus compared to state 5. The more occurrence in state 1 and the fewer occurrence in state 5 during the resting-state may reflect the effort in children with attention problems, who cannot sufficiently compensate for the frontal FC deficit during cognitive tasks [81]. Findings in ADHD individuals provide evidence to support this speculation by showing that the frontal gyrus exhibited significantly increased node efficiency during the resting-state in ADHD children [82].

The psychiatric relevance of DFC states can also be interpreted within the concepts of functional integration and segregation [83, 84]. Functional integration reflects the interaction of different modules to orchestrate information transmission from one to the other. In contrast, functional segregation reflects that the information processing is isolated for each module. Increased functional segregation has been observed in numerous mental diseases [82, 85, 86], characterized by a deficit in sustained attention. The DFC state characterized by strongly positive within-network FC and negative between-network FC might reflect high segregation between networks. On the other hand, state comprised weak within-network FC and relatively larger between-network FC, especially between SM and VS networks, which can be interpreted as increased functional integration. Therefore, we speculate that children spending more time in the functionally segregated state and less time in the functionally integrated state can be a potential cause that leads to a decreased capacity for sustained attention.

Further, the mediation analyses show that attention problems and ADHD symptoms mediate the effect of DFC states on cognitive performance. The attentional process is essential for the formation basis of cognitive development [87]. Various investigations have pointed out that individual differences in early attentional development predict later cognitive functioning [88]. The highly informative behavioral measure of infant attention can also provide insight into memory in early development [89]. In addition, alterations of attentional processes possibly contribute to cognitive deficits in many brain diseases [90–93]. Part of what was found in our study was that the occurrence of the DFC states was associated with both the attention problem and cognition. This is of interest because the DFC patterns have previously been implicated in attention during task conditions [94]. Our finding indicates the covariance between DFC states and cognition is partially related to psychiatric problems, especially attention problems. A possible hypothesis is that if a child’s brain is not well developed as reflected in more occurrences in a functionally segregated state and fewer occurrences in a functionally integrated state, then attention problems will occur, which might impact cognitive performance.

### Future Direction

A potential drawback of our mediation analysis is the cross-sectional nature [53, 95]. The mediation analysis used in our work does not assume causal aspects of these relationships due to the potential for bias in cross-sectional mediational models, but just to discover how much the covariance between DFC states and cognition can be explained by mental health. The ABCD data used in this study contains only two sessions which might not be appropriate for comprehensively exploring the longitudinal changes in DFC and children’s phenotypes and their causal pathway (e.g., DFC->attention problem->cognitive performance). In future studies, when more ABCD data are available for multiple time points, we can build a comprehensive longitudinal changes model to investigate developments in DFC and phenotypic measures and their causal relationships.

We noted that across all the association results in the present study, the highest correlation was r = 0.1150. Although previous brain-wide association studies have typically reported higher correlations (r > 0.2), our reported effect sizes are consistent with previous findings based on the ABCD data, where the documented effects of brain-wide associations are around 0.1 [37, 96]. We argued that previously higher correlations can be due to inflation by chance in small samples [97]. Two independent population subsamples can reach the opposite results of brain-behavior associations solely due to sampling variability in small sample sizes [96]. Recent reports have indicated that the small correlations with phenotypic measures might explain only a small variance [97–99]. Such a small effect size might be due to the heterogeneity of the general population. In that case, the one-for-all model does not work properly. In future studies with multimodal brain characteristics, it is worth dividing the whole sample from the general population into appropriate subgroups, each with a more homogeneous brain representation.

## Figures and Tables

**Table 1:** Basic demographics of subjects

Basic Demographics	Baseline	Second-year
**Total Subject**	10988	3622
**Age** (month)	119.01 ± 7.50	143.20 ± 7.57
**Gender** (F/M)	5269/5719	1667/1955
**Height** (inch)	55.26 ± 3.32	60.08 ± 3.78
**Weight** (Ibs)	82.58 ± 23.74	106.65 ± 31.41
**Race** (W/B/H/A/O/unknown)	5756/1619/2239/223/1149/2	2082/389/729/67/355/0
**Cognition** (nihtbx_totalcomp)	86.44 ± 9.00	88.20 ± 9.73
**Psychiatric problem** (CBCL attention)	2.94 ± 3.46	2.68 ± 3.28
**Psychiatric problem** (CBCL ADHD-DSM-5)	2.59 ± 2.95	2.28 ± 2.77

F = Female; M = Male; W = White; B = Black; H = Hispanic; A = Asian; O = others; NIHTBX = National Institutes of Health Cognition Battery Toolbox; CBCL = Parent-Child-Behavior-Checklist-Scores.

## Data Availability

The code of Neuromark framework has been integrated in the group ICA Toolbox (GIFT, https://trendscenter.org/software/gift/). The ABCD data used in the present study can be accessed upon application from NDA (https://nda.nih.gov/). Other MATLAB codes of this study can be obtained from the corresponding author.

## Abbreviations

ABCD: Adolescent Brain Cognitive Development
ADHD: Attention Deficit Hyperactivity Disorder
AUD: auditory network
CB: cerebellum network
CC: cognitive control network
DFC: dynamic functional connectivity
DM: default-mode network
DSM: Diagnostic and statistical manual of mental disorders
FC: functional connectivity
FDR: False discovery rate
fMRI: functional magnetic resonance imaging
ICA: independent component analysis
LMM: linear mixed-effect model
MNI: Montreal Neurological Institute
QC: quality control
ROIs: regions of interest
SC: subcortical network
SM: sensorimotor network
TC: time-course
TDCCS: Toolbox Dimensional Change Card Sort Task
TFT: Toolbox Flanker Task
TLSWMT: Toolbox List Sorting Working Memory Test
TORRT: Toolbox Oral Reading Recognition Task
TPCPST: Toolbox Pattern Comparison Processing Speed Test
TPSMT: Toolbox Picture Sequence Memory Test
TPVT: Toolbox Picture Vocabulary Task
TR: repetition time
VS: visual network
